# Effects of FTY720 on Lung Injury Induced by Hindlimb Ischemia Reperfusion in Rats

**DOI:** 10.1155/2017/5301312

**Published:** 2017-11-09

**Authors:** Liangrong Wang, Feifei Chen, Yafei Pan, Lina Lin, Xiangqing Xiong

**Affiliations:** Department of Anesthesiology, The First Affiliated Hospital of Wenzhou Medical University, Wenzhou, China

## Abstract

**Background:**

Sphingosine-1-phosphate (S1P) is a biologically active lysophospholipid mediator involved in modulating inflammatory process. We investigated the effects of FTY720, a structural analogue of S1P after phosphorylation, on lung injury induced by hindlimb ischemia reperfusion (IR) in rats.

**Methods:**

Fifty Sprague-Dawley rats were divided into groups SM, IR, F3, F5, and F10. Group SM received sham operation, and bilateral hindlimb IR was established in group IR. The rats in groups F3, F5, and F10 were pretreated with 3, 5, and 10 mg/kg/d FTY720 for 7 days before IR. S1P lyase (S1PL), sphingosine kinase (SphK) 1, and SphK2 mRNA expressions, wet/dry weight (W/D), and polymorphonuclear/alveolus (P/A) in lung tissues were detected, and the lung injury score was evaluated.

**Results:**

W/D, P/A, and mRNA expressions of S1PL, SphK1, and SphK2 were higher in group IR than in group SM, while these were decreased in both groups F5 and F10 as compared to IR (*p* < 0.05). The lung tissue presented severe lesions in group IR, which were attenuated in groups F5 and F10 with lower lung injury scores than in group IR (*p* < 0.05).

**Conclusions:**

FTY720 pretreatment could attenuate lung injury induced by hindlimb IR by modulating S1P metabolism and decreasing pulmonary neutrophil infiltration.

## 1. Introduction

Limb ischemia reperfusion (IR) injury is commonly presented in restoration of blood flow to previously hypoxic extremities following severe crush injury, vessel damage, or some surgical procedures. Limb IR injury is well demonstrated to result in various local and systemic effects, where the latter could include multiple organ failure ultimately leading to death [1, 2]. Lung tissue is very vulnerable to activated neutrophils and oxygen free radicals stimulated by limb IR [3], and lung injury is characterized by leukocyte infiltration, lung capillary barrier damage, pulmonary epithelial cell swelling, and apoptosis [4, 5]. However, the mechanisms involved in this process are complicated; accumulating evidence supports the critical role of polymorphonuclear (PMN) in the development of lung injury [6]. Therefore, strategies for limiting pulmonary PMN sequestration could serve as a promising alternative therapy to attenuate lung injury induced by limb IR.

Sphingosine-1-phosphate (S1P) is a biologically active lysophospholipid that is primarily produced by both sphingosine kinase 1 (SphK1) and sphingosine kinase 2 (SphK2) from sphingosine in response to various cellular stimuli [7], while its degradation is mainly mediated by S1P lyase (S1PL). S1PL, a pyridoxal phosphate-dependent enzyme, could irreversibly cleave S1P into ethanolamine phosphate and trans-2-hexadecenal maintaining S1P homeostasis. It has been now suggested that S1P could mediate diverse biological processes by acting on its receptors in an autocrine and paracrine manner. Functioning as an intracellular second messenger, S1P is associated with cellular proliferation, lymphocyte emigration, vascular endothelial reorganization, and apoptosis [8]. Moreover, increasing number of studies has demonstrated that S1P plays some roles in organ IR injury [9, 10]. FTY720 (fingolimod), a prodrug with promising immunoregulatory effect, is a structural analogue of S1P and could be phosphorylated by Sphk2 into FTY720-P. The phosphorylated product FTY720-P could bind to S1P receptors type 1, type 3, type 4, and type 5 and reduce lymphocyte cell trafficking by lymphocyte egress from lymphoid tissues such as lymph nodes and thymus [11]. It has been documented that FTY720 could protect organs against IR injury [12–15], and one of the potential mechanisms by which FTY720 may have exerted its protective effects was associated with its ability to reduce neutrophil priming [12]. However, the effect of FTY720 on lung injury remote to hindlimb IR remains unknown.

Thus, the purpose of the present study was to evaluate the treatment of FTY720 on lung injury induced by hindlimb IR in rats; histological changes, pulmonary water content, and blood gas analysis were used to assess lung injury; the PMN/alveolus ratio indicated neutrophil infiltration in lung tissue; also, the mRNA expressions of S1PL, SphK1, and SphK2 were measured.

## 2. Materials and Methods

### 2.1. Animal Preparation

All experiments were performed in accordance with the guidelines of the National Institutes of Health Guide for the Care and Use of Laboratory Animals. All experiment procedures were approved by the Animal Ethics Committee of Wenzhou Medical University.

Adult healthy male Sprague Dawley (SD) rats weighing 300 to 350 g were obtained from the Animal Experimental Center of Wenzhou Medical College. Animals were kept under standard conditions and had free access to standard rat chow and water ad libitum until the morning of the experiment. All rats were anesthetized with intraperitoneal administration of 90 mg/kg ketamine and 1 mg/kg acepromazine and placed in the supine position. Tracheotomy was established using a 15-gauge catheter, and a polyethylene catheter was inserted into the left carotid artery for direct blood pressure monitoring and blood gas sampling. A caudal vein was cannulated with a 7-gauge IV catheter for receiving an intravenous infusion of Ringer's solution (5 mL·kg^−1^·h^−1^). Bilateral hindlimb ischemia was induced by applying rubber band tourniquets high around each thigh, and ischemia was verified by disappearance of blood flow signal with Doppler blood flow meter (ES-1000SPM, Hadeco Company, Japan). At the end of the 3-hour ischemia, reperfusion of the limbs was achieved by releasing the tourniquets and blood flow signal was regained.

### 2.2. Experimental Design

Fifty rats were divided into 5 groups, 10 in each group:

1. Group SM: the rats were treated with 2 mL distilled water lavage for 7 consecutive days before the tourniquet was put in place around the hindlimb without inflation.
2. Group IR: the rats were treated with 2 mL distilled water lavage for 7 consecutive days before hindlimb ischemia. Here, hindlimb IR model was established by 3-hour bilateral lower limb ischemia using tourniquets followed by reperfusion up to 3 h.
3. Group F3: the rats were treated with FTY720 (3 mg/kg/d, dissolved in 2 mL distilled water) for 7 consecutive days before hindlimb ischemia.
4. Group F5: the rats were preconditioned with FTY720 (5 mg/kg/d, dissolved in 2 mL distilled water) for 7 consecutive days before hindlimb ischemia.
5. Group F10: the rats were pretreated with FTY720 (10 mg/kg/d, dissolved in 2 mL distilled water) lavage for 7 consecutive days before hindlimb ischemia.

### 2.3. Blood Gas Analysis

Arterial blood gases were analyzed immediately using i-STAT Portable Clinical Analyzer (i-STAT Corporation, East Windsor, New Jersey) before ischemia initiation and at the end of reperfusion, respectively.

### 2.4. Measurement of S1PL, SphK1, and SphK2 mRNA by Real-Time PCR

The right lower lung lobe tissues were homogenized and total RNA was extracted using TRIzol reagent (Shanghai Sangon Biological Engineering Technology & Services, Shanghai, China) according to the manufacturer's instructions. For real-time PCR, 1 *μ*g of the total RNA from each sample was resuspended in 25 *μ*L final volume of reaction buffer and cDNA was reverse transcribed using a synthesis kit. Housekeeping genes glyceraldehyde-3-phosphate dehydrogenase (GAPDH), *β*-actin, and *β*2 microglobulin were used for data normalization. Relative expression levels of mRNA were determined using the 2^−ΔΔCt^ method. The primers used for real-time PCR were listed below: S1PL forward primer 5′-GCA TCT ACG CAT CTC CAA-3′, reverse primer 5′-GCA ACC ATC TTC CTG TCA-3′; SphK1 forward primer 5′- TGG ACT TGG AGA GTG AGA A-3′, reverse primer 5′-CAG AGG AAC GAG GTA TGT G-3′, SphK2 forward primer 5′-GCT CCT ATT GGT CAA TCC TT-3′, reverse primer 5′-TGT CGT TCT GTC TGT ATG AG-3′, GAPDH forward primer 5′-AAT GCA TCC TGC ACC AA-3′, reverse primer 5′-GTA GCC ATA TTC ATT GTC ATA-3′, *β*-actin forward primer 5′-GAA GAT CAA GAT CAT TGC TCC T-3′, reverse primer 5′-TAC TCC TGC TTG CTG ATC CA-3′, and *β*2 microglobulin forward primer 5′-ATG GGA AGC CGA ACA TAC TG-3′ and reverse primer 5′-CAG TCT CAG TGG GGG TGA AT-3′.

### 2.5. Histological Assessment in Lung Tissue

The left lower lung lobe was fixed in 4% buffered formalin solution overnight at room temperature, dehydrated and embedded in paraplast, and then 4 *μ*m-thick sections were obtained. Lung histopathology was examined under a light microscope after hematoxylin and eosin (HE) staining by a pathologist who was blinded to the group allocation. The degree of lung damage was assessed using a modified histological scoring system described before in our previous study [16].

### 2.6. Measurement of PMN Infiltration

The PMN/alveolus ratio (P/A) was used to assess the degree of PMN infiltration. We counted PMNs and alveoli per high-power field (HPF, 400x) in ten randomly selected areas of each sample, and the PMN/alveolus ratio (P/A) was calculated.

### 2.7. Lung Water Content Evaluation

Freshly harvested left upper lung lobe samples were weighed to obtain “wet” weight, then placed in an oven for 24 h at 60°C and weighed again to obtain “dry” weight. The wet/dry weight ratio (W/D) was determined to indicate water content in lung tissue.

### 2.8. Statistical Analysis

Statistical analysis was performed with SPSS version 15.0 (SPSS, Chicago, IL). Data were analyzed for normality with the Kolmogorov-Smirnov method, and the normally distributed data were expressed as mean ± SD. To compare normally distributed data between each group, one-way ANOVA followed by the Bonferroni post hoc test was employed. A *p* value < 0.05 was considered significant.

## 3. Results

### 3.1. The Changes in Arterial Blood Gas Parameters

The baseline arterial blood gas parameters, including pH, arterial partial pressure of oxygen (PaO_2_), arterial partial pressure of carbon dioxide (PaCO_2_), and base excess (BE), among these five groups, were comparable (data not shown). At the end of reperfusion, pH, PaO_2_, and BE were significantly lower (*p* < 0.05), whereas PaCO_2_ was higher in group IR than in group SM (*p* < 0.05; Table 1). As compared to group IR, PaCO_2_ was significantly lower (*p* = 0.014) and pH, PaO_2,_ and BE were higher in group F10 (*p* < 0.05; Table 1).

### 3.2. Lung W/D and P/A Ratios

**Figure 1:**
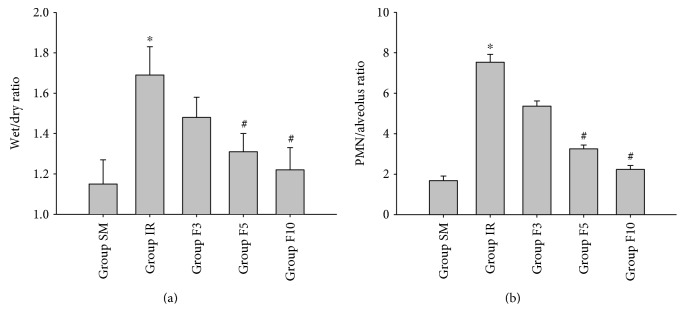
Lung wet/dry weight ratio and PMN/alveolus ratio. Rats were divided into five groups: groups SM, IR, F3, F5, and F10. The rats in group SM received sham operation and bilateral limb IR (ischemia 3 h/reperfusion 3 h) in group IR. The rats in groups F3, F5, and F10 were treated with FTY720 3 mg/kg/d, 5 mg/kg/d, and 10 mg/kg/d, respectively, for 7 consecutive days before limb IR. Upon 3 h reperfusion, wet/dry weight ratio (W/D) and PMN/alveolus ratio (P/A) were calculated. (a) wet/dry weight ratio; (b) PMN/alveolus ratio. ^∗^*p* < 0.05 versus group SM; ^#^*p* < 0.05 versus group IR.

As shown in Figure 1, lung W/D and P/A ratios in group SM were low, which were significantly higher in group IR (*p* < 0.05). Additionally, lung W/D and P/A ratios in both group F5 and group F10, but not in group F3, were significantly increased as compared to group IR (*p* < 0.05; Figures 1(a) and 1(b)).

### 3.3. The Expressions of S1PL, SphK1, and SphK2 mRNA in Lung Tissues

**Figure 2:**
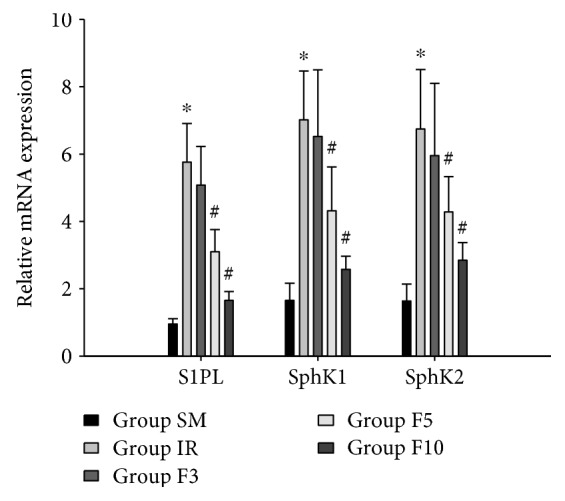
The expressions of S1PL, SphK1, and SphK2 mRNA in lung tissues. Rats were divided into five groups: groups SM, IR, F3, F5, and F10. The rats in group SM received sham operation and bilateral limb IR (ischemia 3 h/reperfusion 3 h) in group IR. The rats in groups F3, F5, and F10 were treated with FTY720 3 mg/kg/d, 5 mg/kg/d, and 10 mg/kg/d, respectively, for 7 consecutive days before limb IR. Upon 3 h reperfusion, the expressions of S1PL, SphK1, and SphK2 mRNA were measured. S1PL: S1P lyase; SphK1: sphingosine kinase-1; SphK2: sphingosine kinase-2. ^∗^*p* < 0.05 versus group SM; ^#^*p* < 0.05 versus group IR.

Compared to their low levels in group SM, the mRNA expressions of S1PL, SphK1, and SphK2 were significantly upregulated in group IR (*p* < 0.05; Figure 2). As expected, the mRNA expressions of S1PL, SphK1, and SphK2 were downregulated in both group F5 and group F10 as compared to group IR (*p* < 0.05; Figure 2); no significant differences in there mRNA expressions were found between group F3 and group IR (*p* > 0.05).

### 3.4. The Assessment of Histological Changes in Lung Tissues

**Figure 3:**
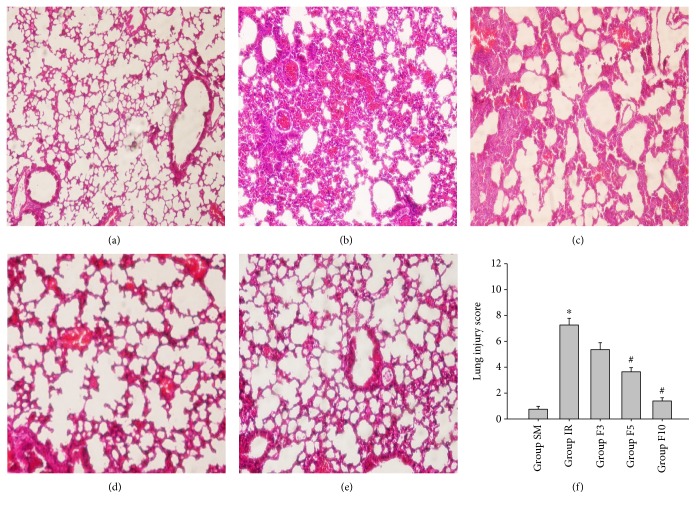
Histological changes in lung tissues. Rats were divided into five groups: groups SM, IR, F3, F5, and F10. The rats in group SM received sham operation and bilateral limb IR (ischemia 3 h/reperfusion 3 h) in group IR. The rats in groups F3, F5, and F10 were treated with FTY720 3 mg/kg/d, 5 mg/kg/d, and 10 mg/kg/d, respectively, for 7 consecutive days before limb IR. Upon 3 h reperfusion, lung damage was examined under a light microscope and the modified histological scoring system was evaluated. (a) Group SM; (b) group IR; (c) group F3; (d) group F5; (e) group F10; (f) histological scores. ^∗^*p* < 0.05 versus group SM; ^#^*p* < 0.05 versus group IR.

No obvious histological abnormalities in lung tissue were noticed in group SM (Figure 3(a)). The lung presented severe lesions in group IR (Figure 3(b)); light microscope examination revealed lung interstitial edema, neutrophil infiltration, alveolar architecture destruction, and intra-alveolar hemorrhage. Treatment with a larger dose of FTY720 significantly reduced these histological damages (Figures 3(d) and 3(e)), which were also evidenced by decreased lung injury scores in both group F5 and group F10 as compared to group IR (*p* < 0.05; Figure 3(f)).

## 4. Discussion

The main findings of our study were that (1) pretreatment with FTY720 had beneficial effects in attenuating lung injury induced by hindlimb IR and that (2) these effects might be mediated by modulating S1P metabolism and inhibition of neutrophil infiltration in the lung tissue.

Various models are reported to simulate ischemic muscle damage, which includes the application of a tourniquet, femoral artery clamping, and iliac artery clamping. Among these, tourniquet application is the only method proven to produce complete ischemia with no residual flow [17]. During arterial occlusion of the lower limb, the tissue supplied by the occluded artery may suffer varying degrees of ischemic injury. Further complications appear during reperfusion, not only locally in necrosis and microcirculatory damage but also in remote organs such as the lung [18]. In the present study, we demonstrated that FTY720, which becomes a structural analogue of S1P following phosphorylation, could exert protective effects in alleviating lung injury induced by bilateral hindlimb IR, which was evidenced by decreased lung water content, improved oxygenation, and reduced histological injury.

Sphingosine 1-phosphate (S1P), a bioactive phospholipid, is produced by the phosphorylation of sphingosine through the action of both SphK1 and SphK2. Binding of S1P to its receptors triggers a wide array of immunological events, such as disrupting lymphocyte trafficking [19], modulating inflammatory cytokine production [20], and inhibition of apoptosis [21]. The roles of S1P signaling in modulating inflammatory response in lung tissue are complicated. Increased vascular permeability is commonly found in lung injury induced by hindlimb IR, while plasma S1P is reported to regulate the vascular tone and maintain the vascular integrity [22]. Similarly, the protective effects of S1P and FTY720 in limiting pulmonary vascular leakage were proved in a murine model of LPS-induced acute lung injury [22]. Conversely, in a dose-dependent manner, the systemic administration of S1P can induce bronchial hyperresponsiveness, increased lung resistance, and proinflammatory cytokines in mice [23], but intrapulmonary application of FTY720 and SphK inhibitors could attenuate experimental asthma acting as an antiasthmatic drug [24]. The discrepancy may attribute to the different pathogenesis including various cell types involved.

SphK1 is critical in modulating inflammatory processes, which could be upregulated in several conditions. The inhibition of SphK1 expression could suppress neutrophil activation and decrease lung permeability and cytokine formation [25], and its protective effects had been elaborated in allergic asthma and sepsis-induced inflammation [26]. FTY720 is now considered as a competitive inhibitor of SphK1 [27], while some showed that FTY720 could inhibit the activities of both SphK1 and SphK2 and suppress the phosphorylation of sphingosine by SphKs, resulting in decreased S1P production [28]. FTY720 itself is a prodrug and unable to inhibit lymphocyte emigration. It is increasingly known that SphK-2, but not SphK1, mediates the phosphorylation of FTY720 into FTY720-p [28], which is responsible for its mechanism of a wide range of immunomodulatory effects through various S1P receptor types except type 2 [29]. S1PL is the only enzyme that leads to irreversible degradation of S1P to phosphoethanolamine and corresponding fatty aldehydes, and the latter can be further metabolized into fatty acids and/or alcohols [30]. Intratracheal LPS led to enhanced pulmonary S1PL expression and decreased S1P level in mice, while inhibition of S1PL activity caused an elevated S1P level and decreased neutrophils priming in lung tissue. FTY720 could not be catabolized by S1PL, but instead inhibited the activity of S1PL without affecting gene and protein expressions in mice or cell lines [25, 31]. Our results revealed that a relatively low dose of FTY720 (3 mg/kg/d, for 7 consecutive days) had no significant effects on S1PL mRNA expression but could be downregulated when the dose increased to 5 or 10 mg/kg/d. Moreover, we demonstrated that hindlimb IR resulted in a remarkable increase in mRNA expressions of SphK1 and 2; both of the SphK mRNA expressions were downregulated by a relatively large dose of FTY720 (5 and 10 mg/kg/d but not 3 mg/kg/d, for 7 days). In summary, treatment with FTY720 led to inhibition of S1PL and SphK mRNA levels, suggesting that the effects of FTY720, at least partially, depend on its action on S1P metabolism.

Although its exact underlying mechanisms were undefined, many studies have shown that pulmonary neutrophil sequestration plays a vital role in lung injury caused by hindlimb IR [32]. Recruited neutrophils in the lung tissue are activated, leading to abundant production of reactive oxygen species, proteases, and proinflammatory cytokines. Reactive oxygen species, in turn, also induces the expression of adhesion molecules on the microvessel endothelium and facilitates transendothelial migration of neutrophils; these would subsequently cause lung injury [32]. Our study demonstrated that the lung damage and infiltration of neutrophils were obviously attenuated by FTY720 preconditioning. FTY720, a S1P receptor agonist, can cause sequestration of lymphocytes into within peripheral and mesenteric lymph nodes, producing a significant decrease in circulating lymphocytes [33]. Conversely, the effects of FTY720 on neutrophil infiltration are controversial. FTY720 treatment can markedly decrease neutrophil infiltration, vascular permeability, and peripheral blood lymphocyte counts in the mouse kidney subject to IR injury [34]. In a rat transplant model, FTY720 (0.5 mg/kg) pretreatment had been shown to rescue isografts and allografts from posttransplant preservation/reperfusion injury, with lower tubular damage scores [35]. Although circulating neutrophils were unchanged, neutrophil infiltration and IL-1 were significantly reduced [35]. When FTY720 dose reaches 1 mg/kg in cats, the number of circulating neutrophils could be also reduced [36]. Moreover, depletion of CD4 T cells was demonstrated to improve liver function and reduce neutrophil infiltration in mouse liver IR injury [37]. These findings suggested a possible lymphocyte-dependent mechanism in neutrophil infiltration, and FTY720 is capable to reduce neutrophil infiltration in IR injury. In contrast, several studies suggested that neutrophil infiltration in kidney and liver IR injury was not affected by treatment with FTY720 [13–15]. Particularly, treatment with FTY720 has been demonstrated to reduce mortality and prevented secretion of proinflammatory cytokines in the lung, liver, and kidney, whereas no effect on myeloperoxidase content, an indicator for neutrophil infiltration, was detected by treatment with FTY720 [38]. However, 48-hour reperfusion itself in the abovementioned study had no effect on MPO content. Here, we established IR injury of 3-hour ischemia of bilateral hindlimb ischemia followed by 3-hour reperfusion, and our findings suggested that FTY720 inhibited neutrophil infiltration and could be considered as a potential therapy in hindlimb IR injury through limiting the inflammatory response. We assumed that the duration of the ischemia and reperfusion phase might contribute to the differences. Additionally, three different preconditioning doses of FTY720 lavage in rats were measured in our study, and a dose-dependent effect of FTY720 was found.

There are several limitations to our study. First, FTY720 was chosen because we acknowledged the critical role of S1P signaling in modulating inflammation and attempted to investigate the possible therapy, but FTY720 is just one of various S1P receptor agonists; the effects of other S1P receptor agonists in attenuating lung inflammation were not evaluated. Second, the long-term effects of FTY720 on lung injury and survival of rats remained unknown. Finally, we did not detect S1P levels due to technical unavailability.

In conclusion, through modulating S1P metabolism and inhibiting pulmonary neutrophil infiltration, pretreatment with FTY720 could attenuate lung injury induced by hindlimb IR.

## Figures and Tables

**Table 1:** Arterial blood gases at the end of reperfusion ($\bar{x}\pm s$, *n* = 10).

	Group SM	Group IR	Group F3	Group F5	Group F10
pH	7.36 ± 0.16	7.18 ± 0.11^∗^	7.24 ± 0.09	7.30 ± 0.12	7.35 ± 0.15^#^
PaO_2_ (mmHg)	260 ± 54	148 ± 44^∗^	185 ± 20	228 ± 53	253 ± 58^#^
PaCO_2_ (mmHg)	45 ± 8	70 ± 10^∗^	62 ± 6	51 ± 6	48 ± 5^#^
BE (mM)	−2.9 ± 1.2	−9.9 ± 2.1^∗^	−7.2 ± 1.5	−5.9 ± 1.2	−3.5 ± 1.3^#^

Rats were divided into five groups: groups SM, IR, F3, F5, and F10. The rats in group SM received sham operation and bilateral limb IR (ischemia 3 h/reperfusion 3 h) in group IR. The rats in groups F3, F5, and F10 were treated with FTY720 3 mg/kg/d, 5 mg/kg/d, and 10 mg/kg/d, respectively, for 7 consecutive days before limb IR. Upon 3 h reperfusion, arterial blood gases were analyzed. PaO_2_: arterial partial pressure of oxygen; PaCO_2_: arterial partial pressure of carbon dioxide; BE: base excess. ^∗^*p* < 0.05 versus group SM; ^#^*p* < 0.05 versus group IR.
